# Incidence and Factors Associated with Burnout in Anesthesiology: A Systematic Review

**DOI:** 10.1155/2017/8648925

**Published:** 2017-11-28

**Authors:** Filippo Sanfilippo, Alberto Noto, Grazia Foresta, Cristina Santonocito, Gaetano J. Palumbo, Antonio Arcadipane, Dirk M. Maybauer, Marc O. Maybauer

**Affiliations:** ^1^Department of Anesthesia and Intensive Care, IRCCS-ISMETT (Istituto Mediterraneo per i Trapianti e Terapie ad Alta Specializzazione), Via Tricomi 5, 90127 Palermo, Italy; ^2^Department of Anaesthesia and Intensive Care, Policlinico Universitario G. Martino, University of Messina, Messina, Italy; ^3^Department of Biopathology, Medical and Forensic Biotechnologies (DIBIMEF), Section of Anesthesiology, Analgesia, Emergency and Intensive Care, Policlinico “P. Giaccone”, University of Palermo, Via del Vespro 129, 90100 Palermo, Italy; ^4^School of Anaesthesia and Intensive Care, University of Catania, Catania, Italy; ^5^Critical Care Research Group, Prince Charles Hospital, The University of Queensland, Brisbane, QLD, Australia; ^6^Department of Anaesthesiology and Intensive Care, Philipps University, Marburg, Germany; ^7^Cardiothoracic Anaesthesia and Intensive Care, Manchester Royal Infirmary, Central Manchester University Hospitals NHS Foundation Trust, University of Manchester and Manchester Academic Health Science Centre, Manchester, UK

## Abstract

**Background:**

Burnout syndrome has reached epidemic levels among physicians (reported around 50%). Anesthesiology is among the most stressful medical disciplines but there is paucity of literature as compared with others. Analysis of burnout is essential because it is associated with safety and quality of care. We summarize evidence on burnout in anesthesiology.

**Methods:**

We conducted a systematic review (MEDLINE up to 30.06.2017). We included studies reporting burnout in anesthesiology with no restriction on role or screening test used.

**Results:**

Fifteen surveys/studies described burnout in anesthesiology, including different workers profiles (nurses, residents, consultants, and directors). All studies used the Maslach Burnout Inventory test but with significant differences for risk stratification. Burnout prevalence greatly varied across studies (10%–41% high risk, up to 59% at least moderate risk). Factors most consistently associated with burnout were strained working pattern, working as younger consultant, and having children. There was no consistent relationship between burnout and hospital characteristics, gender, or marital status.

**Conclusions:**

Burnout prevalence among anesthesiologists is relatively high across career stages, and some risk factors are reported frequently. However, the small number of studies as well as the large differences in their methodology and in reporting approach warrants further research in this field.

## 1. Introduction

Several medical disciplines are deemed at high risk of psychological stress [1]. On the one hand, stress could exert beneficial effects when faced with a proper cognitive approach and adequate coping strategies [2]. Indeed, physician's ability to tackle challenging situations may increase self-confidence and enhance the sense of well-being and of being helpful [3]. On the other hand, an exaggerated degree of stress may lead to decreased satisfaction, undermining physician's mental and physical health [4–7] and finally leading to a psychological syndrome known as burnout [8]. This syndrome is reaching epidemic levels in the medical population with prevalence reported around or over 50% [9]. Burnout develops in response to chronic interpersonal stressors [10] and is more likely in the absence of appropriate support from healthcare organization and managers [11–13], and its presence affects negatively patients' care [14–16] and physicians' professionalism [17–20].

It is broadly accepted that the three main components of burnout syndrome are high emotional exhaustion (EE), high depersonalization (DP), and low personal accomplishment (PA) [21]. In brief, EE is a subjective work-related sense of fatigue, DP is a defense mechanism in the attempt to separate oneself from work, and low PA represents a feeling of frustration with work-related achievements. Burnout syndrome differs from depression because it is specific to the work environment. The presence of burnout has been associated with the relationship impairment between team members [22], all together with a decreased work activity [23] and a worsened quality of care delivered [18, 24, 25], possibly increasing healthcare costs [26]. It has been hypothesized that this can create a vicious circle, fostering more trenchant cost-containment policies, which in turn may increase stress perception of the staff members [23]. Furthermore, burnout also plays a role in the development of major depression or substance abuse [27]. With this background, it is easy to understand why physician's burnout may seriously affect the healthcare worker performances and well-being, with an impact on the level of patient's care. Therefore, it is not surprising that strategies adopted to reduce its impact are of increasing interest and under scrutiny [28].

Anesthesiology is certainly one of the most stressful medical disciplines, daily exposing physicians to high responsibilities and stressful situations such as the management of life-threatening scenarios. Moreover, the work pattern may be perceived as more stressful than other medical disciplines since duties include overnight shifts as well as weekends and festivities. Although it would not be surprising to find a high incidence of burnout among healthcare professionals working as anesthesiologists, a systematic assessment has not been conducted as yet. Therefore, we conducted a systematic search aiming at exploring the incidence of burnout in anesthesiology and possibly describing its characteristics according to working place, career state and progression, and private characteristics (i.e., family support), in order to provide a general perspective of burnout in anesthesia practitioners.

## 2. Materials and Methods

We undertook a systematic web-based advanced literature search through the* National Health Service (NHS) Library Evidence* tool on the incidence of burnout in anesthesiology. We followed the approach suggested by the PRISMA statement for reporting systematic reviews [30]. However, we expected a difficult comparison and aggregation of data concerning burnout, since there is a wide range of measure tools for burnout in the literature and the response rate in the survey is usually suboptimal. Moreover, we expected that most of the studies had a descriptive design, not aiming at comparing different medical disciplines. For such reasons we a priori discarded the idea of performing a meta-analysis and/or pooling data for getting the overall incidence in anesthesiology. Yet, the aim of this expanded systematic search is to provide a broader insight of the topic.

In order to identify relevant articles, an initial computerized search of MEDLINE (PubMed) was conducted from inception until 30 January 2017 and with its findings we started the manuscript drafting. The search was then updated before the submission (final search conducted on the 30 June 2017) and the manuscript was amended accordingly.

Our core search was structured in the combination of terms obtained from two groups. The first group included only the term “burnout”; the second group included the following words: “anaesthesia”, “anesthesia”, “anaesthesiology” and “anesthesiology”. Inclusion criteria were prespecified according to the PICOS approach (Table 1). Study selection for determining the eligibility for inclusion in the systematic review and data extraction from the selected studies were performed independently by three reviewers (FS, GF, and AA). Discordances were resolved by involving two other authors (CS, MOM) and/or by consensus.

Language restrictions were applied: we read the full manuscript only for articles published in English, German, French, or Italian. A manual search was conducted independently by all the authors, exploring the list of references of the findings of the systematic search. We excluded book chapters, reviews, editorials, and letters to the editor from the qualitative synthesis.

## 3. Results

**Figure 1:**
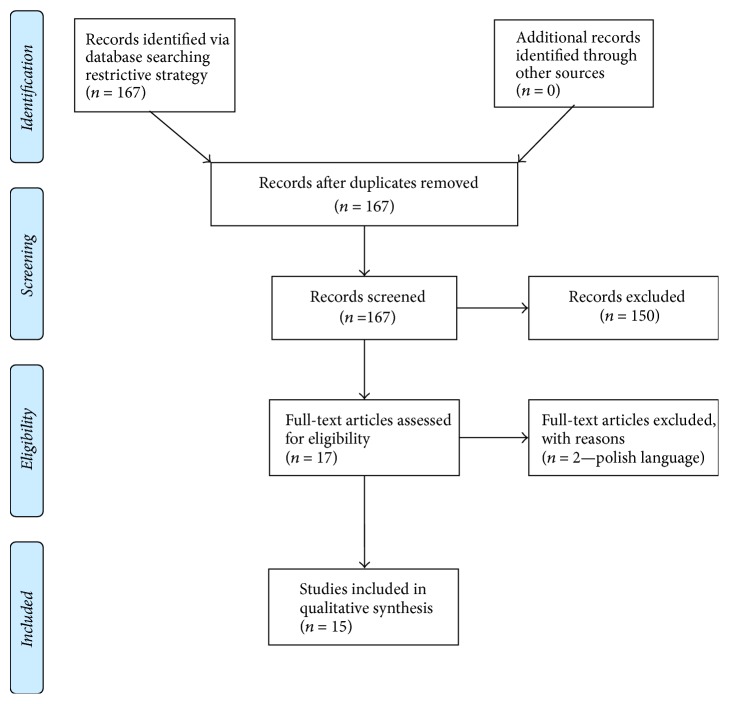
Adapted from [29]. For more information, visit http://www.prisma-statement.org.

The literature web-based search produced a total of 167 hits. Reviews and editorials were excluded, and seventeen studies investigated the selected topic according to the prespecified PICOS criteria; we finally included 15 findings for the qualitative synthesis, excluding two articles because they are published in Polish language [31, 32] (see Figure 1).

Of these findings, two manuscripts investigated burnout among residents/trainees only [33, 34] and three among attendings/consultants only [25, 35, 36], and five included both categories [37–41]. Three other studies mixed a population of anesthesiologists and anesthesia nurses [42–44], while the other two focused on anesthesiologists in leading position (directors of anesthesia or anesthesia program directors) [45, 46].

A summary of the main findings on burnout risk is reported in Table 2. All the studies used the Maslach Burnout Inventory (MBI) test to investigate burnout. The MBI test has been used in the full 22-items (*n* = 9) [25, 35–37, 39, 40, 42–44] or in shorter versions (*n* = 3 used the short version with 12 questions [33, 45, 46], *n* = 1 used only the nine questions of the EE subscale [38], and *n* = 2 utilized the Dutch MBI version,* Utrechtse Burnout Schaal*-MBI [34, 41]).

All factors studied for their association (positive, neutral, or negative) towards the development of burnout in anesthesiology are summarized in Table 3.

## 4. Discussion

Physician's burnout is an emerging problem for healthcare systems with an increase in the percentage of physicians reporting at least one burnout symptom, from 45% to 54% between 2011 and 2014 [9]. Burnout is particularly common in healthcare professionals working in the emergency/critical care field, as shown by the Medscape physician lifestyle report 2016 [47], with the highest percentage of burnout occurring in critical care and emergency medicine physicians (55%), closely followed by anesthesiologists (50%). It is possible to tackle burnout and a large recent meta-analysis found that individual-focused and structural or organizational strategies could result in significant reductions in overall burnout among physicians with a decrease of about 10% [28]. Therefore, efforts in amelioration of work quality are mandatory for preventing burnout.

In our systematic review, we aimed at describing figures of burnout in the field of anesthesiology and we found that this topic has been investigated at different levels, involving nurses, residents, fully certified anesthesiologists, and also chiefs/directors. Even if the risk of burnout in anesthesiologists is real, it should be considered that, in a study conducted in Australia, anesthesiologists showed subscale scores of burnout in the lower range (high EE 20%, high DP 20%, and low PA 37%) as compared with other historical medical groups [25]. On the other side, Nyssen et al. showed a mean stress level in Belgian anesthetists of 51%, which anyway is no higher than burnout levels found in other working settings and slightly lower than average figures reported by a recent meta-analysis [28]. More integrated research between medical disciplines is needed to determine whether anesthesiology has or has not a high risk of burnout, and it should be considered that differences between healthcare systems and organizations are likely to affect the findings.

Burnout negatively affects the healthcare systems at different levels, and more importantly it is associated with poorer patient safety [15] and quality of care, since it may manifest through fatigue and reduced cognitive functioning, finally influencing individual work performance and resulting in higher risk of errors [14–16, 48]. This is particularly true for anesthesiologists, whose underperformance may play a significant role in the genesis of patient harm with catastrophic outcomes as well as malpractice claims [48]. In a study on US residents, De Oliveira Jr. et al. found an association between burnout (± depression) and lower quality of care delivered (more medication errors and/or a lower rate of “best practice” in anesthesiology) [33]. On the other hand, when asking patients about their satisfaction with regard to anesthesia, Capuzzo et al. did not find any relationship with staff burnout [42].

Our study illustrates a wide variation of burnout prevalence rates among anesthesiologists across all career stages of the discipline, from residents/trainees to chiefs/directors. This is not surprising since anesthesiology is one of the most stressful medical disciplines. Anesthesiologists carry high responsibilities during surgery and may frequently face stressful scenarios such as management of unanticipated difficult airways, cardiac arrest, and other life-threatening emergencies. Moreover, the work pattern, at least for larger hospitals, may also be perceived as more stressful due to high numbers of on-calls [49] and night shifts and the higher possibility of working during weekends and festivities determining an imbalance between personal and professional lives [13], compared to other physicians category. The influence of strained work pattern on anesthesiologists' burnout is manifest in several studies. De Oliveira Jr. et al. reported that high workload (70 hours/week) is associated with a higher incidence of burnout and depression [33]; similarly, Rui et al. showed that working over 60 hours/week is independently associated with higher DP and lower PA [39]. Nyssen et al. reported that the main sources of stress described by anesthesiologists were the lack of control overtime management, their busy work plan, the complexity of clinical tasks, and the huge sense of clinical responsibility [38]. Likewise, Portuguese anesthesiologists indicated that the main factors generating stress were strained professional relationships, unskilled leadership by superiors, work overload, indiscipline of surgeons, lack of adequate working conditions (e.g., inappropriate resources and lack of nursing staff), and technically challenging situations (e.g., airway management) [36]. Indeed Brazilian anesthesiologists reported that working night shifts was the most frequent feature of anesthesiologists classified with burnout syndrome [35]. We believe that a work-related predisposing condition in the field of anesthesiology may be due to the reduced interaction with patients and their families. In turn, this may enhance a sensation of low rewards and recognition for the contribution of anesthesia staff to patient's care. Nonetheless, this hypothesis is highly speculative and higher degree of burnout has been described in intensive care physicians [47], despite a similar background (in many countries joint training program of anesthesia and intensive care) and higher interaction with patients and their families. Other factors such as therapeutic obstinacy and provision of futile care may at least partially explain the higher burnout in intensive care [50].

While the work pattern and the time management seem related to burnout, an interesting finding of our systematic search is that there is no clear relationship between burnout and hospital characteristics. The analysis of literature seems indeed controversial, with a Dutch study reporting no differences between anesthesiologists working at academic centers as compared with those working in community hospitals [41]. On the other hand, Downey et al. found lower PA in anesthesiologists with academic practice as compared to those working in the private settings [37], and Rui et al. showed a higher risk of burnout in anesthesiologists working in urban and larger scales hospitals [39]. It is possible that anesthesiologists working with academic degree may be exposed to a higher incidence of burnout; indeed, the group of De Oliveira Jr. conducted two studies on burnout in Chairmen of academic anesthesia departments and in anesthesia program directors [45, 46]. In both studies, the authors found that an at-least moderate risk of burnout was present in over 70% of the respondents [45, 46], and high risk was found in 20% [46] to 28% [45]. Importantly, in both studies about one in four respondents reported high likelihood of stepping down from the job within the following two years (22% to 28%). A high score of EE and DP as well as low PA (range 56%–62%) is described by Shams and El-Masry in a mixed population prevalently consisting of academic anesthesiologists, and almost 70% of the surveyed population reported job stress [40]. A national US survey investigating wellness among academic anesthesiology chairs showed their strong interest towards burnout counseling, which scored forth among 22 different wellness topics (after conflict management, dealing with adverse events training and stress management) [51]. On the other side, Rui et al. showed that Anesthesiology Directorship in China seems an independent protective factor against burnout [39].

With regard to the gender, one study reported that females were more exposed to burnout [33, 43], while another showed no association between sex and burnout [41], and three studies found higher burnout risk in men [35, 40, 52]. We found some concordance on the influence of age on burnout, with younger staff reported at higher risk in most of the retrieved studies [36, 38, 40, 43, 52], although one study found the highest risk in the age range of 30–50 years and being over 10 years in the profession [35]. Only one study reported a possible protection from burnout in younger anesthesiologists, with those starting practice within the previous 5 years found to have lower DP [37]. In the context of age and experience in the anesthesiology profession, one study showed higher burnout risk in anesthesiologists as compared with residents (20% versus 11%, resp.) with higher DP in younger consultants (5 to 15 years in anesthesiology practice) without relationship with age [41]. There are several possible explanations for the influence of age or experience on burnout. Anesthesiologists with lower experience may suffer from feeling a higher degree of responsibility when staring their work as consultants, but they also may feel stressed by (or unsecure in) managing difficult scenarios like difficult airways, cardiac arrest, and other emergencies on their own. Other possible reasons for the burnout peak during the initial stages of anesthesiology career could be the result of a “surviving effect,” as shown by Downey et al., where older anesthesiologists have lower EE and DP [37]. Indeed excessive stress either may cause an early quit of anesthesiology career by those not able to tolerate it or may encourage the implementation of effective coping strategies in those continuing in the profession. These responses to stress have been already described among emergency physicians [53]. Some studies explored how active intervention could decrease burnout occurrence; Sussman et al. elegantly showed that a transition to a better on-call model may produce positive effects on residents and may even improve patient's care [54]. The authors assessed the impact of transitioning from a 24-hour to a 16-hour on-call model among Canadian anesthesiology residents, demonstrating that a change to shorter on-call improved significantly resident wellness and reduced burnout. Moreover, residents described less fatigue and more satisfaction with their educational experience. Importantly, when asked about the impact of the new on-call model with regard to patients' safety, only 2% of the residents thought that safety was worse with the new model, and most residents affirmed that it had improved or remained unchanged (both 49%) since the implementation of the shorter on-call. Lower satisfaction with organization has been associated with EE in one study [36], and in another study respondents reported “work organization” as the most frequent problem [41]. It is likely that professional role is more important than age, which just correlates with personal life, for instance, being married and having children. In this regard, marital status was found not associated with burnout in three studies [33, 35, 52], while only one found such association [40]. On the other hand, an association between burnout and parental status has been described by three studies, with having children being a risk factor [35, 36, 40]. A possible interpretation for this aspect could be the difficulty of coping with familiar duties while exposed to a significant workload.

### 4.1. What Physicians and Organization Can Do about Burnout

Although this is not the target topic of our systematic review, we briefly describe what measures can be undertaken to reduce burnout incidence which is primarily driven by workplace stressors [55]. In order to promote the well-being of themselves and of their patients, physician and healthcare organization have the duty to put in place efforts to decrease the risk of burnout. Grossly, interventions for decreasing burnout can be divided into two main categories, those physician-directed (targeting individuals) and the organization-directed ones (targeting working environment) [56, 57].

Physician-directed interventions typically involve courses on mindfulness or cognitive behavioral techniques that help enhancing job competence and improving communication skills and coping strategies.

Organization-directed interventions usually involve changes in schedule with reductions in the workload intensity and/or an increase in the level of participation in decision-making. Other cases involve changes to the policies and practice with the aim of improving teamwork, changing the approach to work evaluation, and increasing the supervision. In this way, physicians may feel a reduction in job demand and enhancement regarding their job control.

In a recent meta-analysis looking at controlled interventions to reduce burnout in physicians, Panagioti et al. [58] found that majority of interventions were physician-directed (60%), including mindfulness-based stress reduction techniques, educational interventions targeting physicians' self-confidence, and communication skills or a combination of these. Within the category of organization-directed interventions, the authors found both studies with simple workload interventions focusing on rescheduling shifts and reducing workload and studies with broader interventions incorporating also multidisciplinary meetings to enhance teamwork and leadership. This meta-analysis showed that organization-directed interventions were associated with higher treatment effects compared with physician-directed interventions, but also physicians-targeted interventions produced a significant (though small) benefit, reducing burnout. With regard to the career stage, interventions on more experienced physicians showed evidence of greater effectiveness compared with interventions targeting less experienced physicians [58].

While it is difficult to draw firm conclusions since the studies included in the meta-analysis differed in terms of content and length of interventions, study design and quality, and length of follow-up, it seems reasonable that both physician and healthcare organization promote courses and interventions aimed at decreasing the incidence of physician's burnout.

### 4.2. Limitations

Our systematic review cannot be considered as conclusive on burnout in anesthesiology staff and we found several issues in interpreting the results of these studies. Fifteen studies reported the burnout in anesthesia staff and all used either the full MBI questionnaire or one of its shorter forms; however, the high heterogeneity of their methods warrants high caution in looking at prevalence of burnout and even more in drawing conclusions. Indeed, the results should be interpreted not only in the context of the different country and healthcare system where anesthesia staff was surveyed, but carefully looking at the cut-offs of MBI used for defying the risk of burnout as well. In general, we found different MBI cut-offs for high risk of burnout used across the retrieved studies, ranging from >23 [44] to >29 [38, 43] for EE, from >8 [35, 44] to >14 [36] for DP, and from <24 [36] to <34 [35, 43] for PA. Interestingly, even within the same group of authors we found discrepancies. Indeed, the active group of De Oliveira Jr. et al. conducted three studies reporting the incidence of burnout in anesthesiology residents, in Chairmen of academic anesthesiology departments and in anesthesiology program directors. It was striking for us to find that the authors used different cut-offs for high risk of DP (>9 [45] or >12 [33, 45]). Moreover, in one of their survey the definition of moderate risk of burnout relies on precise scoring in each of the three subscales (but without reporting incidence of moderate risk [33]), while in two other studies the authors prefer to use a combination of the results of the three subscales [45, 46].

In view of these limitations, we believe that it would be meaningless to argue about the results of the retrieved studies in the attempt to find a reliable prevalence of burnout in anesthesiology. Indeed, excluding a very small study that focused on residents of several medical disciplines and found no burnout in a sample of 13 anesthesia residents [34], the remaining papers report the incidence of high-risk burnout with a range from 10% [35] to 41% [33]. The same findings are clear when looking at incidence of high risk of burnout according to results of single subscales (see Table 2). Here, the ranges of anesthesiology staff scoring at high risk of burnout were 16%–62% for high EE [40, 43], 10%–91% for high DP [36, 37], and 13%–65% for low PA [37, 43].

Therefore, more research is certainly desirable on burnout in anesthesiology, and probably in other medical disciplines, in order to clarify whether anesthesia puts staff at higher risk as compared with other medical disciplines. From our systematic review, it seems reasonable to refer to the MBI (possibly in its full version) as it is the most frequently used method for burnout assessment; however, it urges that cut-offs are carefully checked in order to keep consistency in results reporting.

## 5. Conclusions

The prevalence of burnout syndrome among anesthesiologists is relatively high, and it seems higher in younger physicians with lower experience. Other consistently described risk factors, associated with anesthesiologists' burnout, are occupational conditions (mostly work overload and/or being young consultant) as well as personal circumstances (having children). However, the small number of studies included as well as the large differences in their methodology and in reporting approach warrants further research in this field.

## Figures and Tables

**Table 1:** “PICOS” approach for selecting studies in the systematic search.

PICOS	Characteristics of studies included for the systematic search
*(1) Participants*	Anesthesiology staff at any career stage (residents, consultants, directors, and nurses)
*(2) Intervention*	Assessment of burnout syndrome
*(3) Comparison*	None
*(4) Outcomes*	Risk of burnout syndrome evaluated either overall or according to subscales of burnout
*(5) Study design*	Prospective surveys

**Table 2:** Studies included in the systematic search on burnout in anesthesia. For each study, we quote in the second column the population investigated, the response rate, the questionnaire used for assessment of burnout, and the country where the survey was performed. Burnout prevalence is indicated either overall or according to subscales, as reported by the author. In the last column, we report the criteria adopted by each study in order to define the risk of burnout. EE: emotional exhaustion; DP: depersonalization; and PA: personal achievements. MBI: Maslach Burnout Inventory. UBOS: Utrechtse Burnout Schaal.

Study	Population Response rate Burnout questionnaire used Country/region	Burnout	Subscales scores	Criteria for burnout classification
Kluger et al. Anaesthesia 2003	Anesthesiologists 422/700 (60%) Full MBI (22q: EE 9, DP 5, PA 8) Australia	-	EE high 20%; moderate-high 43% DP high 20%; moderate-high 47% PA low 37%; moderate-low 63%	Burnout was defined as EE (>27) combined with DP > 10 and/or PA < 40. Moderate risk of burnout not reported

Nyssen et al. Brit J Anaesth 2003	Anesthesiologists & anesthesia residents 151/318 (48%) MBI (only EE 9q) Belgium (French speaking)	-	EE high 40%; moderate-high 84%	High risk (severe) of burnout if EE > 29, moderate risk if EE = 18–29. Used a Likert scale 1–7 rather than 0–6

Morais et al. Eur J Anaesthesiol 2006	Anesthesiologists 270/850 (31%) Full MBI (22q: EE 9, DP 5, PA 8) Portugal	-	EE high 58% DP high 91% PA low 45%	EE: high risk if >26 DP: high risk if >14 PA: high risk if <24

Prins et al. Int J Behav Med 2007	Residents, multiple disciplines (13/29 anesthesia) 158/292 (54%) UBOS-MBI (20q,8 EE,5 DP,7 PA) Netherlands (Groningen)	High 4% Moderate 9% (overall); Anesthesia 0%	-	High risk (severe) of burnout if >28 on EE + >10 on DP; or if >28 on EE and <24 on PA

Capuzzo et al. Anesth Analg 2007	43 anesthesiologists & 68 anesthesia nurses (79%) Full MBI (22q– n/r EE, DP, PA) Italy (4 centers, Tuscany)	High 27%	-	High risk of burnout if EE > 26 and DP > 9 and/or PA < 34

Chiron et al. J Health Psycol 2010	74 anesthesiologists & 77 anesthesia nurses (78% overall) Full MBI (22q: EE 9, DP 5, PA 8) France (8 centers, n/r)	-	EE high 16%; moderate-high 38% DP high 18%; moderate-high 41% PA low 13%; moderate-low 46%	EE: high risk if >29, moderate risk if 18–29 DP: high risk if >11, moderate risk if 6–11 PA: high risk if <34, moderate risk if 34–39

De Oliveira et al. J Clin Anesth 2011	Anesthesia program directors 100/132 (76%) Short MBI (12q: EE 5, DP 3, PA 4) US	High 20% Mod.-high 30% Moderate 22%	-	Scores calculated as proportional values of the full MBI. EE: high risk if >26; DP: high risk if >9; PA: high risk if <32. High risk of burnout if high EE and DP and low PA. Moderate risk criteria n/r

De Oliveira Jr. et al. Anestheisology 2011	Chairs of academic anesthesia Dept. 102/117 (79%) Short MBI (12q: EE 5, DP 3, PA 4) US	High 28% Mod.-high 31% Moderate 11%	-	Scores proportional to the full MBI. EE: high risk if >26; DP: high risk if >12; PA: high risk if <32. High risk of burnout if high EE and DP and low PA. Moderate risk if at least 2/3 met

Downey et al. Anaesthesia 2012	Anesthesiologists, anesthesia residents, and fellows 57/61 (93%) Full MBI (22q: EE 9, DP 5, PA 8) US (Boston)	-	EE high 30%; moderate-high 61% DP high 10%; moderate-high 32% PA low 65%; moderate-low 93%	EE: high risk if >26, moderate risk if 17–26 DP: high risk if >12, moderate risk if 7–12 PA: high risk if <32, moderate risk if 32–38

De Oliveira Jr. et al. Anaesth Analg 2013	Anesthesia residents 1508/2773 (54%) Short MBI (12q: EE 5, DP 3, PA 4) US	High 41%	-	Scores calculated as proportional values of the full MBI EE: high risk if >26, moderate risk if 17–26 DP: high risk if >12, moderate risk if 7–12 PA: high risk if <32, moderate risk if 32–38 High risk if moderate or high score in at least 2/3 of subscales

Orena et al. Saudi J Anaesth 2013	12 anesthesiologists & 6 anesthesia nurses (78% overall) Full MBI (22q – n/r EE, DP, PA) Italy (Milan)	-	EE high 22%; moderate-high 39% DP high 22%; moderate-high 44% PA low 17%; moderate-low 33%	EE: high risk if >23, moderate risk if 15–23 DP: high risk if >8, moderate risk if 4–8 PA: high risk if <30, moderate risk if 30–36

Shams and El-Masry Sultan Qaboos Univ Med J 2013	Academic anesthesiologists, anesthesia residents 98/134 (73%) Full MBI (22q – n/r EE, DP, PA) Egypt (Mansoura)	-	EE high 62% DP high 56% PA low 58%	Not clearly reported

Magalhães et al. Rev Bras Anestesiol 2015	Anesthesiologists (12% in leading position) 134/241 (56%) Full MBI (22q: EE 9, DP 5, PA 8) Brazil (Federal District)	High 10% Low 8%	EE high 23%; moderate-high 54% DP high 28%; moderate-high 77% PA low 48%; moderate-low 86%	EE: high risk if >25, moderate risk if 16–25 DP: high risk if >8, moderate risk if 3–8 PA: high risk if <34, moderate risk if 34–39

Van der Wal et al. Eur J Anaesthesiol 2016	Anesthesia residents and anesthesiologists of Dutch Society 655/1955 (33%) UBOS-MBI (20q:EE 8, DP 5, PA 7) Netherlands	High 18%	-	High scores defined as above 75th percentile of the UBOS-MBI manual

Rui et al. J Eval Clin Pract 2016	Anesthesiologists, residents, and specialty doctors 395/416 (95%) Full MBI (22q: EE 9, DP 5, PA 8) China		EE high 15%; moderate-high 40% DP high 8%; moderate-high 25% PA low 35%; moderate-low 66%	EE: high risk if >26, moderate risk if 16–26 DP: high risk if >12, moderate risk if 6–12 PA: high risk if <32, moderate risk if 32–39 High EE (≥27), high DP (≥13), and low PA (≤39) scores indicated high degrees of burnout

**Table 3:** Summary of factors identified as associated, neutral, and protective towards the development of burnout in anesthesiology.

Factor	Associated with burnout	Neutral effect on burnout	Protective effect on burnout
Younger age	Morais et al., Eur J Anaesthesiol 2006 Shams and El-Masry, Sultan Qaboos Univ Med J 2013 Nyssen et al., Br J Anaesth 2003 Abut et al., Saudi J Anaesth 2012 Chiron et al., J Helth Psychol 2010	Magalhães et al. Rev Bras Anestesiol 2015	Downey et al., Middle East J Anaesthesiol 2013

Female gender	De Oliveira Jr. et al., Anesth Analg 2013 Chiron et al., J Helth Psychol 2010 Goldhagen, Adv Med Educ Pract 2015	Van Der Wal et al., Eur J Anaesthesiol 2016	Magalhães et al. Rev Bras Anestesiol 2015 Abut et al., Saudi J Anaesth 2012 Shams and El-Masry, Sultan Qaboos Univ Med J 2013

Higher career stage	Van Der Wal et al., Eur J Anaesthesiol 2016 Morais et al., Eur J Anaesthesiol 2006		Rui et al., J Eval Clin Prct 2016 Shams and El-Masry, Sultan Qaboos Univ Med J 2013 Chiron et al., J Helth Psychol 2010

High workload	De Oliveira Jr. et al., Anesth Analg 2013 Morais et al., Eur J Anaesthesiol 2006 Magalhães et al. Rev Bras Anestesiol 2015 Sussman and Paul, Adv Med Edu Pract 2015 Rui et al., J Eval Clin Prct 2016 Nyssen et al., Br J Anaesth 2003 Downey et al., Middle East J Anaesthesiol 2013		

Marital status	Shams and El-Masry, Sultan Qaboos Univ Med J 2013	De Oliveira Jr. et al., Anesth Analg 2013 Magalhães et al., Rev Bras Anestesiol 2015 Abut et al., Saudi J Anaesth 2012 Morais et al., Eur J Anaesthesiol 2006	

Parental status	Abut et al., Saudi J Anaesth 2012 Morais et al., Eur J Anaesthesiol 2006 (females)	Morais et al., Eur J Anaesthesiol 2006 (males)	

Low support from family	De Oliveira Jr. et al., J Clin Anesth 2011 De Oliveira Jr. et al., Anesthesiology 2011		

Supervision/job support			Shams and El-Masry, Sultan Qaboos Univ Med J 2013 De Oliveira Jr. et al., Anesth Analg 2015

Academic practice	Downey et al., Middle East J Anaesthesiol 2013 De Oliveira Jr. et al., J Clin Anesth 2011 De Oliveira Jr. et al., Anesthesiology 2011	Morais et al., Eur J Anaesthesiol 2006	Rui et al., J Eval Clin Prct 2016 Morais et al., Eur J Anaesthesiol 2006

Alcohol consumption	De Oliveira Jr. et al., Anesth Analg 2013		
